# DR-CoT: dynamic recursive chain of thought with meta reasoning for parameter efficient models

**DOI:** 10.1038/s41598-025-18622-6

**Published:** 2025-10-06

**Authors:** Aarush Sinha, OmKumar Chandra Umakanthan, Sudhakaran Gajendran

**Affiliations:** 1 School of Computer Science and Engineering (SCOPE), Vellore Institute of Technology-Chennai, Kelambakkam - Vandalur Road, Chennai, Tamil Nadu 600127 India; 2 School of Electronics Engineering (SENSE), Vellore Institute of Technology-Chennai, Kelambakkam - Vandalur Road, Chennai, Tamil Nadu 600127 India

**Keywords:** CoT, Reasoning, LLMs, BERT, Computational models, Computational neuroscience

## Abstract

Chain-of-Thought (CoT) prompting has revolutionized reasoning in Large Language Models (LLMs), enabling them to tackle complex tasks by mimicking step-by-step human thought processes. However, traditional CoT methods often suffer from high computational costs and context dilution, limiting their effectiveness, particularly in resource-constrained or real-time applications. To address these challenges, we introduce **Dynamic Recursive Chain-of-Thought (DR-CoT)**, a novel reasoning framework for parameter-efficient models. DR-CoT synergistically integrates recursive reasoning, dynamic context truncation, and a voting mechanism. By selectively retaining the most salient context within a fixed token budget and aggregating inferences from multiple independent reasoning chains, DR-CoT significantly enhances reasoning accuracy. Extensive evaluations on challenging reasoning benchmarks, including GPQA Diamond and AIME2024, demonstrate the efficacy of DR-CoT. On GPQA Diamond, DR-CoT sees Pass@1 accuracy gains of 1.5% for Gemini 2.0 Flash Thinking Experimental, 2.7% for Grok 3 Beta, and 4.4% for o3 Mini. Similarly, AIME2024 results reveal consistent improvements of 3-4 percentage points across evaluated models. Furthermore, DR-CoT enhances zero-shot classification performance on GPQA Diamond, enabling compact BERT-sized models to surpass larger language models such as GPT-4 and LLaMA 2. In code generation tasks using HumanEval, DRCoT empowers models to exceed the performance of established frontier LLMs, including LLaMA 70B, Phi-3, and Claude Sonnet. These comprehensive results underscore DR-CoT’s effectiveness in bridging the performance gap between parameter-efficient models and state-of-the-art LLMs across multiple domains.

## Introduction

Recent breakthroughs in natural language processing (NLP)^1–3^ have showcased the exceptional capabilities of large language models (LLMs), including LLaMA3^4^, GPT-4^5^, and GPT-3.5^6^, in reasoning using chain-of-thought (CoT) prompting. These models demonstrate capabilities of breaking down complex tasks into simple sequential steps. Nevertheless, these models are computationally expensive or require inference costs in any sort of setting.

Conversely, smaller models provide enhanced computational efficiency but frequently falter on complex reasoning tasks due to constrained representational capabilities and suboptimal context management. Our Dynamic Recursive Chain-of-Thought (DR-CoT) framework presented in this work specifically targets smaller and more efficient models. Integrating recursive reasoning with dynamic context truncation, enables smaller models to selectively preserve the most relevant information.

Our method distinctively enables smaller models to execute complex reasoning tasks through systematic refinement of intermediate outcomes and dynamic context management, effectively reducing the performance disparity between computationally efficient models and their larger counterparts. The subsequent sections present the DR-CoT framework in detail and provide comprehensive evaluations demonstrating its effectiveness across diverse challenging benchmarks.

### Our contributions

The main contributions of this work can be summarized as follows:

1. *Dynamic Recursive Reasoning* We propose the DR-CoT framework that integrates recursive reasoning into the chain-of-thought paradigm, enabling the model to adaptively manage and refine its intermediate reasoning steps.
2. *Dynamic Context Truncation* We develop a dynamic context truncation method that preserves the most relevant reasoning steps while ensuring that the context remains within predefined token limits, thus balancing efficiency and informativeness.
3. *Voting Mechanism* We introduce a voting mechanism that aggregates outputs from multiple independent reasoning chains to determine the most accurate final answer, thereby mitigating errors from any single chain.

**Fig. 1:**
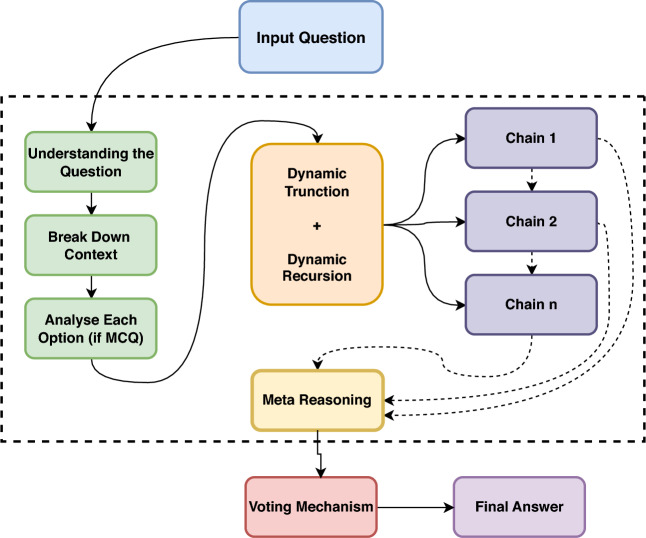
Overview of the entire process and architecture of our framework.

In this paper, we provide an overview of the key sections. Section 2 presents the related work, discussing existing literature and prior studies on Chain of Thought reasoning. Section 3 details the proposed methodology, with each subsection describing a specific component of our approach also detailed in the Fig. 1. Section 4 reports the experimental results across various models and benchmarks. Section 5 provides a detailed error analysis of our framework. Section 7 concludes the paper, summarizing our findings and discussing potential future directions. Section 8 outlines future avenues for our research. Section 9 outlines the limitations of our work.

## Related work

The Scratchpad approach^7^ pioneered CoT by introducing intermediate computations, enabling language models to decompose complex problems into manageable parts. This improved coherence and reasoning accuracy, laying the groundwork for future developments in prompting techniques for large-language models (LLMs). Prompt tuning enhanced LLMs’ structured reasoning capabilities^8^. Simple prompts like “Let’s think step by step”^9^ also improved reasoning in zero-shot settings. These findings led to research on CoT techniques, including recursion and task decomposition, leading to advancements such as Active Prompting^10^ and Plan-and-Solve^11^.

Chain-of-Knowledge (CoK) Prompting^12^ incorporates external knowledge into the reasoning process, ensuring more reliable and factual sound outcomes. Task decomposition remains crucial, with approaches such as Decomposed Prompting^13^ and Successive Prompting^14^ breaking down complex tasks into manageable sub-problems. Recent explorations into recursion within CoT frameworks have utilized specialized tokens for iterative reasoning^15^. This technique facilitates repeated evaluations of intermediate results, leading to higher accuracy and deeper understanding of complex problems. This recursion-based CoT method has shown particular success in tackling challenging datasets.

Multi-stage CoT prompting has also been explored for domain specific tasks such as detecting and correcting medical errors from clinical notes^16^. It incorporates clinical context learning to enhance performance in zero-shot scenarios, showcasing the adaptability of CoT methods in healthcare settings. CoT reasoning has also been explored across multiple languages through cross-lingual alignment and task-specific solver prompting^17^ and zero-shot multilingual semantic parsing using CoT prompting to augment multilingual datasets^18^. It aims to overcome language limitations inherent in existing zero-shot prompting techniques. Application of CoT has been used to detect and fix system vulnerabilities^19^. It presents a unified prompting approach that demonstrates significant improvements in identifying, discovering, and patching vulnerabilities, showcasing the effectiveness of CoT in critical software engineering tasks.

Chain-of-Thought (CoT) prompting has expanded beyond text to show promising results in multimodal domains. Image of Thought^20^ introduced a framework that guides Multimodal Large Language Models (MLLMs) through step-by-step visual reasoning processes. This approach helps MLLMs break down complex visual tasks into interpretable reasoning steps, improving performance on visual understanding. In speech translation,^21^ demonstrated that CoT techniques can enhance translation quality. The method decomposes the translation process into discrete steps yielding significant improvements over traditional end-to-end approaches.

CoT reasoning is not always faithful^22^, revealing that models often rationalize their implicit biases, which can lead to logically contradictory answers. Mechanics of long chain-of-thought reasoning^23^, finding that while supervised fine-tuning facilitates the development of extended reasoning chains, reinforcement learning requires careful reward shaping to stabilize the growth of such chains.

Our approach to solving questions systematically follows a structured, multi-layered reasoning chain. While individual components of our Dynamic Recursive Chain-of-Thought (DR-CoT) framework, such as voting mechanisms and context truncation, are established techniques in isolation, the novelty of DR-CoT lies in their synergistic integration within a recursive reasoning process. This framework uniquely combines dynamic context management, recursive refinement of intermediate results, and a voting mechanism to achieve enhanced reasoning capabilities, particularly in resource-constrained environments. The effectiveness of DR-CoT stems from this holistic approach, where each component is carefully orchestrated to address the limitations of traditional Chain-of-Thought methods. The methodology integrates dynamic recursive reasoning, context truncation based on token thresholds, and a voting mechanism to arrive at the most accurate answer. We provide a comparison in Table 1.

**Table 1:** Comparison of DR-CoT with chain-of-thought approaches.

Approach	Key features	Limitations	DR-CoT improvements
Scratchpad^7^	Intermediate computations	Lacks refinement, inefficient context handling	Recursive reasoning for dynamic updates
Simple Prompting^9^	Step-by-step zero-shot prompts	Struggles with complex tasks	Dynamic context truncation, recursion
Plan-and-Solve^10,11^	Task decomposition	Uses a single reasoning chain	Aggregates multiple chains via voting
Chain-of-Knowledge^12^	Integrates external data	Depends on external sources	Manages internal context efficiently
Decomposed Prompting^13,14^	Modular breakdown	Weak error correction	Consensus-based voting reduces errors

## Methodology

**Algorithm 1:**
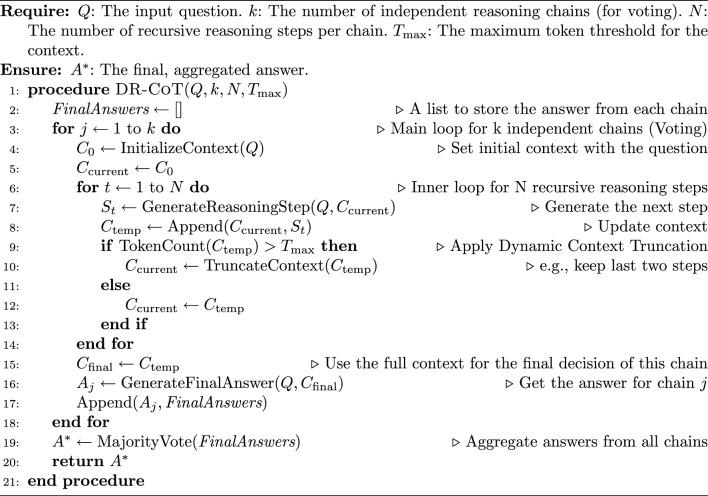
The DR-CoT framework

### Step-by-step reasoning chain

Given a question $Q$ with a set of options $O = \{ o_1, o_2, \ldots , o_n \}$ but **do not include the options if the dataset is a QA dataset**, we apply the following iterative and recursive steps:

1. *Understanding the Question* Identify key concepts, clarify requirements, and outline the objective of $Q$.
2. *Breaking Down the Context* Extract all relevant facts, relationships, constraints, and pivotal elements inherent in the question.
3. *Analyzing Each Option* Examine each option $o_i$ by evaluating supporting evidence and identifying potential contradictions.
4. *Evidence Gathering* Systematically collect and annotate evidence that supports or refutes each $o_i$, ensuring clear logical connections.
5. *Critical Evaluation* Eliminate implausible options through systematic comparison and highlight the most promising candidates.
6. *Final Analysis and Conclusion* Synthesize the gathered insights to justify the selected answer $A^*$ and provide a final explanation.

### Recursive reasoning in DR-CoT

The term “Dynamic Recursive Reasoning” in DR-CoT encapsulates the framework’s ability to iteratively refine its reasoning process via a recursive update mechanism over a dynamically managed context. Formally, let $t$ denote the current iteration, $S_t$ the reasoning step at iteration $t$, and $C_t$ the corresponding context. At each iteration, the model computes:

1 $$\begin{aligned} S_t = F(Q, C_t) \end{aligned}$$

where $Q$ is the original query and $F(\cdot )$ denotes the model-s reasoning function. Immediately after, the context is updated according to:

2 $$\begin{aligned} C_{t+1} = g(C_t, S_t) \end{aligned}$$

Here, the update function $g(\cdot )$ performs dynamic truncation by selecting a subset of $C_t \cup \{S_t\}$ that maximizes relevance–determined via a scoring metric such as semantic similarity or attention weights–while ensuring that the token count does not exceed a predefined threshold $T_{\max }$ (i.e., $|C_{t+1}| \le T_{\max }$).

This recursive update mechanism enables the model to “revisit” and refine its earlier reasoning steps by continuously incorporating only the most pertinent information. Unlike explicit recursive function calls, the recursion here is implicit in the iterative refinement process. Moreover, an efficient implementation can utilize data structures (e.g., priority queues) to sort context elements by relevance, thereby ensuring that the function $g(\cdot )$ operates optimally within the token budget. This combination of dynamic context management and iterative refinement results in a more focused, robust, and computationally efficient reasoning process.

### Dynamic context truncation

To manage reasoning complexity and maintain efficiency, we dynamically truncate the context based on token count. Let $C_t$ represent the context at step $t$ and $T_{max}$ denote the maximum token threshold (e.g., 1800 tokens). The value of $T_{\max }$ is set empirically, chosen as a balance between retaining sufficient context for complex reasoning and respecting the models’ context window limitations and hardware memory constraints. We enforce:

3 $$\begin{aligned} |C_t| \le T_{max}. \end{aligned}$$

This constraint ensures that the model only processes a manageable amount of information at each step, thereby reducing computational overhead and preventing the dilution of important details. By keeping the context within a fixed size, we can maintain efficiency without sacrificing the quality of the reasoning process.

If $|C_t| > T_{max}$, the context is truncated as follows:

4 $$\begin{aligned} C_t' = \{ S_{t-1}, S_{t-2}^{*} \}, \end{aligned}$$

where:

- $S_{t-1}$ is the most recent reasoning step, which captures the latest inference and developments.
- $S_{t-2}^{*}$ is the** most relevant previous step that directly informs the current evaluation**. In this initial implementation, we adopt a simple yet effective heuristic for determining relevance: chronological order within the context window. Specifically, $S_{t-2}^{*}$ is selected as the reasoning step immediately preceding $S_{t-1}$ in the truncated context. This approach assumes that the most recent steps are generally the most pertinent for the current reasoning stage. While more sophisticated relevance metrics, such as attention scores or semantic similarity, could be explored in future work, chronological selection provides a practical starting point to ensure that context truncation retains the most immediately preceding and thus likely most pertinent information for maintaining reasoning flow.

This truncation method allows the model to focus on the most pertinent parts of the reasoning history, ensuring that only critical information is retained for further analysis. For the final reasoning step, the complete context is preserved:

5 $$\begin{aligned} C_{final} = C_t. \end{aligned}$$

Preserving the complete context at the final step guarantees that the final decision is made with full awareness of all the accumulated insights, thereby enhancing the accuracy of the conclusion.

### Voting mechanism

To enhance answer accuracy and mitigate the risk of error from any single reasoning chain, we employ a voting mechanism by running multiple independent reasoning chains. The final answer is determined as follows:

6 $$\begin{aligned} A^* = \mathop {\mathrm {arg\, max}}\limits _{O_i \in \mathcal {O}} \sum _{j=1}^{k} I(A_j = O_i), \end{aligned}$$

where $\mathcal {O}$ represents the set of possible answers, *k* is the number of independent reasoning chains, and the indicator function $I(\cdot )$ is defined as:

7 $$\begin{aligned} I(A_j = O_i) = {\left\{ \begin{array}{ll} 1, & \text {if } A_j = O_i, \\ 0, & \text {otherwise}. \end{array}\right. } \end{aligned}$$

The voting mechanism acts as an ensemble method by aggregating the outputs from multiple reasoning chains. Each chain independently evaluates the problem and proposes an answer, and the indicator function counts how many chains agree on a particular option. The answer with the highest count is then selected as the final answer.

This process significantly reduces the likelihood of random, independent errors, as it prevents any single misstep from dominating the decision-making process. By combining multiple perspectives, the system effectively achieves a consensus, thereby increasing the robustness and reliability of the final decision. This mechanism is particularly useful in complex scenarios where individual reasoning chains might diverge.

However, a key limitation of this approach is its vulnerability to systemic or shared biases within the foundation model. If all reasoning chains are influenced by the same underlying bias, they may collectively converge on the same incorrect answer, and the voting mechanism would fail to correct the error. To mitigate this risk, future work could focus on promoting diversity among the reasoning chains, for instance by using varied prompts or introducing stochasticity through temperature sampling to generate more distinct reasoning paths.

### Meta reasoning

Meta-reasoning enables the model to leverage its reasoning history by maintaining a structured record of prediction paths. At each step *s*, the model receives an instruction template: $I_s =$ “Analyze the question and options thoroughly,” “consider each possibility systematically,” “and produce a step-by-step solution.” For each branching path *t* at step *s*, the agent constructs a reasoning path string:

8 $$\begin{aligned} r_{\text {path}} = \text {''Step x: Reasoning: (confidence: } c_j \text {)''} \end{aligned}$$

This $r_{\text {path}}$ is concatenated with the previous reasoning chain $r_{\text {previous}}$ to form $r_{\text {new}}$:

9 $$\begin{aligned} r_{\text {new}} = \text {concat}(r_{\text {previous}},\, r_{\text {path}},\, ''{\,} '') \end{aligned}$$

This updated chain $r_{\text {new}}$ is then incorporated into the input for the next recursive step:

10 $$\begin{aligned} x_{\text {new}} = \text {tokenize}(x_{\text {previous}},\; ''\text { Reasoning: }'',\; r_{\text {new}}) \end{aligned}$$

Finally, to ensure compatibility with maximum context length, the input is processed via Dynamic Context Truncation described in Sect. 3.3:

11 $$\begin{aligned} x_{\text {processed}} = \text {tokenize}(x_{\text {new}},\; L = C_{final}) \end{aligned}$$

### Why it works?

The effectiveness of the DR-CoT framework stems from the synergistic integration of its core components: recursive reasoning, dynamic context truncation, and an ensemble-style voting mechanism. First, the recursive process allows the model to incrementally refine its reasoning by continuously updating its context with the most relevant intermediate steps, thus reducing noise from less pertinent information. Second, dynamic context truncation ensures that the model operates within resource constraints by discarding extraneous data while preserving critical reasoning cues. Finally, by aggregating outputs from multiple independent reasoning chains via a voting mechanism, DR-CoT mitigates errors from any single chain and converges on a consensus final answer. Together, these mechanisms enable a self-correcting approach to complex problem-solving.

## Results

For LLMs like Gemini2.5 Pro, o3 mini and Grok 3 Beta(Think) we use the available chat interfaces. For the BERT models we utilize a single Tesla 16GB GPU, we also write all our code in PyTorch^24^ and extensively utilize HuggingFace Transformers^25^.

### Reasoning with LLMs

**Fig. 2:**
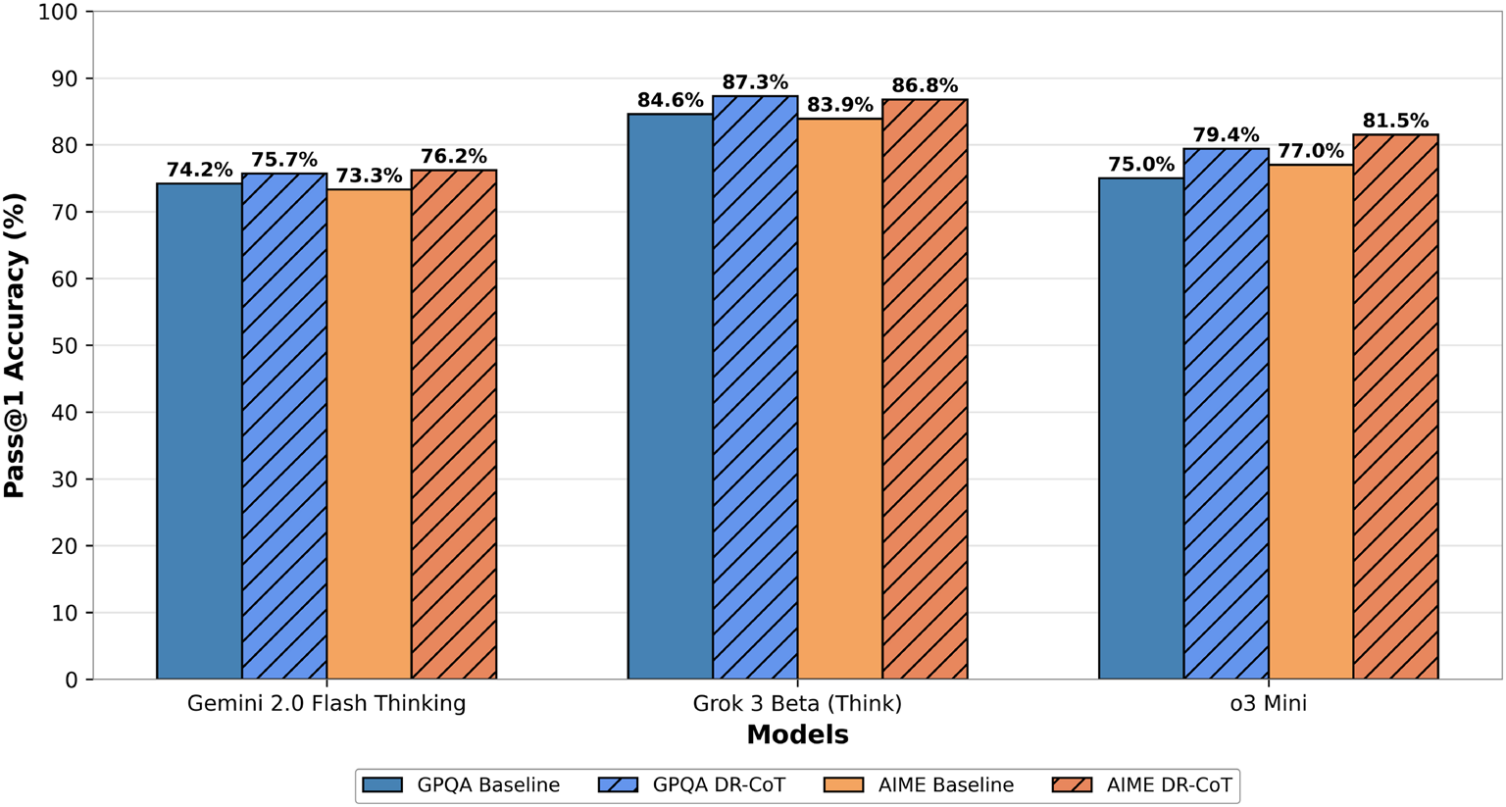
Performance comparison of various models on the GPQA Diamond and AIME2024 datasets, illustrating the improvement achieved with DR-CoT.

Table 2 and Fig. 2 presents a comparative analysis of model performance with and without the Dynamic Recursive Chain of Thought (DR-CoT) framework. We evaluate the Pass@1 accuracy on two challenging datasets: GPQA Diamond^26^ and AIME2024 1. The results consistently demonstrate the effectiveness of DR-CoT in enhancing the reasoning capabilities of large language models.

On the GPQA Diamond dataset, the application of DR-CoT resulted in performance improvements across all tested models. Specifically, Gemini 2.0 Flash Thinking Experimental^27^ achieved an increase from 74.2 to 75.7%, Grok 3 Beta (Think)^28^ improved from 84.6 to 87.3%, and o3 Mini^29^ exhibited the most substantial gain, increasing from 75.0 to 79.4%.

A similar trend was observed on the AIME2024 dataset, where DR-CoT consistently enhanced performance. Gemini 2.0 Flash Thinking Experimental achieved a Pass@1 score of 76.2%, up from 73.3%. Grok 3 Beta (Think) improved from 83.9 to 86.8%, while o3 Mini saw an increase from 77.0 to 81.5%.

**Table 2:** Pass@1 accuracy for GPQA Diamond and AIME2024 benchmarks across frontier models with and without DR-CoT. Bold and italic cells represent the best performing model for each benchmark.

Model	GPQA diamond	AIME2024
Grok 3 Beta (Think)	84.6	83.9
Grok 3 mini Beta (Think)	80.0	***89.5***
DeepSeek-R1	71.5	79.8
Gemini 2.0 Flash Thinking	74.2	73.3
o1	78.0	83.3
o3 mini (high)	79.7	87.3
o3 mini (medium)	76.8	79.6
*Models with DR-CoT Enhancement*		
Grok 3 Beta (Think) + DR-CoT	***87.3***	86.8
o3 mini + DR-CoT	79.4	81.5
Gemini 2.0 Flash Thinking + DR-CoT	75.7	76.2

These results indicate that the integration of DR-CoT provides a significant and consistent boost to model performance across different architectures and datasets illustrated in Fig. 2. The observed improvements underscore the effectiveness of our proposed step-by-step reasoning with adaptive context management, which facilitates enhanced reasoning capabilities in complex problem-solving tasks. Furthermore, the consistent gains across multiple datasets and diverse model architectures highlight the robustness and generalizability of the DR-CoT approach.

### Zero-shot classification

Table 3 presents zero-shot classification accuracy results for various BERT-based models with and without the DR-CoT method on the GPQA Diamond dataset. Our results demonstrate consistent improvements in performance across all models tested.

**Table 3:** Zero-shot classification accuracy on BERT-based models with and without DR-CoT on GPQA Diamond.

Model	Baseline$\uparrow$	Baseline + DR-CoT$\uparrow$
BERT-base^30^	21.2	23.7 (+2.5)
BERT-large	21.2	26.3 (+5.1)
ROBERTa-base^31^	24.7	25.8 (+1.1)
ROBERTa-large	27.3	28.5 (+1.2)
ELECTRA-base^32^	26.2	29.3 (+3.1)
ELECTRA-large	26.3	28.5 (+2.2)
ModernBERT-base^33^	23.7	25.0 (+1.3)
ModernBERT-large	29.8	32.9 (+3.1)

**Table 4:** Performance comparison on GPQA benchmark (Diamond Set and Main Set). Models are grouped by architecture family and sorted by performance within groups. Underline bold highlights the best-performing model with DR-CoT enhancement, while Italic indicates scores that are surpassed by ModernBERT-large with DR-CoT. The highlighted row demonstrates that DR-CoT enables a significantly smaller model to achieve competitive performance against larger, more resource-intensive models.

Model	Diamond Set (%)	Main set (%)
*GPT-4 Models*		
Few-Shot GPT-4	39.3	38.1
Few-Shot CoT GPT-4	38.8	**39.7**
Zero-Shot CoT GPT-4	35.7	39.5
Zero-Shot GPT-4	34.2	32.1
*Smaller Models with DR-CoT Enhancement*		
ModernBERT-large(DR-CoT)	**32.9**	**29.5**
ELECTRA-base(DR-CoT)	29.3	21.4
ELECTRA-large(DR-CoT)	28.5	26.1
ROBERTa-large(DR-CoT)	28.5	23.2
ROBERTa-base(DR-CoT)	25.8	20.1
BERT-large(DR-CoT)	26.3	26.8
ModernBERT-base(DR-CoT)	25.0	23.9
BERT-base(DR-CoT)	23.7	19.4
*GPT-3.5 Models*		
Zero-Shot GPT-3.5-turbo-16k	*30.6*	29.8
Few-Shot CoT GPT-3.5-turbo-16k	*29.6*	*28.0*
Zero-Shot CoT GPT-3.5-turbo-16k	*28.1*	*28.9*
Few-Shot GPT-3.5-turbo-16k	*26.0*	*28.9*
*Llama-2 Models*		
Zero-Shot CoT Llama-2-70B-chat	*31.1*	*28.5*
Few-Shot CoT Llama-2-70B-chat	*28.1*	*29.1*
Zero-Shot Llama-2-70B-chat	*26.5*	*27.6*
Few-Shot Llama-2-70B-chat	*24.0*	*26.9*
*Additional Baselines*		
GPT-4 with search	*27.6*	*28.7*

For BERT-base^30^, accuracy increases from 21.2 to 23.7%, reflecting a gain of 2.5 percentage points, while BERT-large improves significantly from 21.2 to 26.3% (+5.1 percentage points). Similarly, RoBERTa-base^31^ sees a 1.1 percentage point increase, from 24.7 to 25.8%, and RoBERTa-large achieves a gain of 1.2 percentage points, rising from 27.3 to 28.5%. ELECTRA-base^32^ shows a notable improvement, increasing from 26.2 to 29.3% (+3.1 percentage points), while ELECTRA-large sees a gain of 2.2 percentage points, reaching 28.5% from 26.3%. ModernBERT-base^33^ also benefits from DR-CoT, improving from 23.7 to 25.0% (+1.3 percentage points). Notably, the second ModernBERT-large variant demonstrates the highest absolute accuracy, increasing from 29.8 to 32.9%, a gain of 3.1 percentage points.

In Table 4, we compare BERT-sized models paired with DR-CoT against baseline LLMs such as LLaMA 2-70B^34^ and GPT-4^5^. The best-performing model with DR-CoT, ModernBERT-Large, achieves an accuracy of **32.9** and **29.5** on the diamond and main subsets, respectively. Notably, it outperforms significantly larger models models such as GPT3.5, GPT4 and LLama2, demonstrating the effectiveness of our proposed method.

### Code generation

Lastly we test the effectiveness of DR-CoT in code generation tasks. We use HumanEval^35^ as our test bench and apply DR-CoT to two models. The QwenCoder2.5(1B)^36^ and the DeepseekCoder(1.3B)^37^. We evaluate using Pass@1 with the same DR-CoT setup described earlier, modifying only the reasoning steps to better align with coding. Since coding benchmarks include their own test cases, refining answers based on confidence and voting mechanisms becomes irrelevant. To adapt the DR-CoT framework for code generation on the HumanEval benchmark, we implemented a robust candidate selection strategy to maximize the success rate for a Pass@1 evaluation. The goal is to produce a single, correct solution for each problem on the first try. This is achieved through a multi-stage process for each Pass@1 attempt:

### Generation of multiple internal variants

For each problem, our framework generates a portfolio of five distinct *internal variants* to explore different reasoning paths. These are not five independent attempts in the Pass@k sense; rather, they are part of a single, comprehensive generation process. The variants are created using different prompting strategies to ensure diversity:

- *Base Variant* One solution is generated from a general, high-level instruction.
- *Reasoning-Step Variants* Four additional solutions are generated using prompts that are augmented with one of our specific DR-CoT reasoning steps (e.g., “Step 2: Identify potential edge cases...”). This guides the model to focus on different aspects of the problem for each variant.

Different temperature settings are also used to further encourage diverse outputs.

### Syntax validation

All five generated code snippets are first passed through a syntax validator. We use Python’s built-in compile() function to check for SyntaxError. Any variant that fails this check is immediately discarded, as it cannot be executed.

### Sequential evaluation with early exit

This is the core of our Pass@1 strategy. The syntactically valid variants are not evaluated in parallel; they are tested sequentially against the HumanEval test cases. The process is as follows:

1. The framework takes the first syntactically valid variant and runs it against the unit tests within a timeout-controlled subprocess.
2. If this variant passes all tests, the evaluation for this problem stops immediately. The variant is accepted as the successful solution, and the overall Pass@1 attempt is marked as a “pass”. The remaining variants are never tested.
3. If the variant fails the tests (or times out), it is discarded, and the framework moves on to the next syntactically valid variant in the list.
4. This process repeats until a passing solution is found or all valid variants have been tested and have failed.

The reasoning steps are given below:

**Table:** 

Reasoning Steps	
Step 1: Understand the problem statement, input types, and expected output type.	
Step 2: Identify potential edge cases (e.g., empty inputs, zero values, specific constraints).	
Step 3: Outline the core logic or algorithm using pseudocode or comments.	
Step 4: Translate the pseudocode into Python, focusing on clear variable names and structure.	
Step 5: Review the Python code against the requirements and edge cases identified.

**Table 5:** Pass@1 accuracy on HumanEval benchmark for code generation. The table shows standard large language models ranked by performance, with visual indicators showing where DR-CoT-enhanced smaller models would place in the ranking. Gray rows indicate where DR-CoT-enhanced models would rank among larger models, demonstrating how this technique enables smaller models (1.3B-1.5B parameters) to achieve competitive performance with models 10-50x their size.

Model	HumanEval (%)	Rank
*Large Language Models*		
GPT-3.5 (May 2023)	73.2	1
WizardCoder-Python-34B-V1.0	73.2	1
OpenChat-3.5-7B-0106	72.6	3
CodeLlama-70B-Instruct	72.0	4
WhiteRabbitNeo-33B-v1	72.0	4
$\hookrightarrow$ *Qwen2.5Coder-1.5B-Instruct + DR-CoT would rank here (71.4%)*		
Phind-CodeLlama-34B-v2	71.3	6
speechless-coder-ds-6.7B	71.3	6
Magicoder-S-CL-7B	70.7	8
Claude-3-Sonnet (Mar 2024)	70.7	8
Llama3-1.8B-Instruct	69.5	10
Mistral Large (Mar 2024)	69.5	10
Claude-2 (Mar 2024)	69.5	10
Qwen1.5-72B-Chat	68.3	13
Gemini Pro 1.5	68.3	13
StarCoder2-15B-Instruct-v0.1	67.7	15
speechless-starcoder2-15b	67.1	16
Code-290k-6.7B-Instruct	64.6	18
Phi-3-mini-4k-instruct	64.6	18
$\hookrightarrow$ *DeepseekCoder-1.3B-Instruct + DR-CoT would rank here (64.1%)*		
Command-R+	64.0	20
dolphin-2.6-mixtral-8x7b	64.0	20
Gemini Pro 1.0	63.4	22
*Models With DR-CoT*		
Qwen2.5Coder-1.5B-Instruct	54.5	–
Qwen2.5Coder-1.5B-Instruct + **DR-CoT**	**71.4**	($\uparrow$** to 4th)**
DeepseekCoder-1.3B-Instruct	57.3	–
DeepseekCoder-1.3B-Instruct + **DR-CoT**	**64.1**	($\uparrow$** to 18th)**

In Table 5, we highlight the significant impact of incorporating **DR-CoT** on the coding capabilities of smaller language models, as measured by Pass@1 accuracy on HumanEval. Notably, the Qwen2.5Coder-1.5B-Instruct model exhibits a remarkable improvement, increasing from a baseline score of 54.5 to 71.4 when augmented with DR-CoT, a substantial gain ($\Delta = +16.9$). Similarly, the DeepseekCoder-1.3B-Instruct model experiences a significant boost, with its score rising from 57.3 to 64.1, representing a gain ($\Delta = +6.8$). These results underscore the effectiveness of DR-CoT in enhancing the problem-solving and code generation capabilities of these models.

Furthermore, the performance uplift achieved via DR-CoT enables these relatively small models (1.5B and 1.3B parameters) to be highly competitive with, and in some cases outperform, significantly larger models listed in the table 2. For example, Qwen2.5Coder-1.5B-Instruct + DR-CoT (71.4) surpasses much larger models such as Phind-CodeLlama-34B-v2 (71.3), Qwen1.5-72B-Chat (68.3), Mistral Large (69.5), and Gemini Pro 1.5 (68.3). It even approaches the performance of CodeLlama-70B-Instruct (72.0). Likewise, DeepseekCoder-1.3B-Instruct + DR-CoT (64.1) achieves parity with or surpasses models such as Command-R+ (64.0), dolphin-2.6-mixtral-8x7b (64.0), and Gemini Pro 1.0 (63.4). These findings demonstrate that advanced reasoning techniques like DR-CoT can effectively bridge the performance gap, allowing smaller, more efficient models to rival the coding proficiency of significantly larger counterparts.

### Computational overhead

It is important to note that DR-CoT is an inference-time prompting framework and therefore has no impact on a model’s underlying parameter size. The number of parameters for a given model remains unchanged, as DR-CoT does not involve any form of model retraining or fine-tuning.

The primary computational overhead introduced by DR-CoT is an increase in inference time and VRAM usage. The increase in inference time is driven primarily by the voting mechanism, which requires executing $k$ independent reasoning chains. Consequently, the total inference time scales approximately linearly with the number of chains used, representing a direct trade-off between enhanced accuracy and computational cost.

The impact on memory is detailed in Table 6, which presents the VRAM usage of various transformer models with and without the DR-CoT framework. On average, the application of DR-CoT increases VRAM usage by approximately 2.75 GB across all models. The base versions of the models exhibit a lower overall memory footprint compared to their large counterparts, with an average VRAM usage of 4.29 GB without DR-CoT and 7.00 GB with DR-CoT. Meanwhile, large models require significantly more VRAM, averaging 9.4 GB without DR-CoT and 11.6 GB with DR-CoT. The increase in VRAM usage due to DR-CoT varies slightly across models, with the smallest increase observed in ROBERTa-large and ModernBERT-large (2 GB), while other models experience an increase ranging between 2 and 3 GB. Notably, ModernBERT-base exhibits the lowest VRAM usage without DR-CoT (3 GB), whereas ROBERTa-large and ModernBERT-large require the most memory (12 GB) when DR-CoT is applied. These findings suggest that while DR-CoT increases VRAM usage in all models, the relative increase remains within a reasonable range of 2 to 3 GB, making it a feasible enhancement to improve model performance without excessive computational cost.

**Table 6:** Comparison of VRAM Usage with and without DR-CoT, showing absolute and percentage overheads.

Model	VRAM Usage (GB)	VRAM with DR-CoT (GB)	$\Delta$ (GB)	Overhead (%)
BERT-base	4	6	+2	50%
BERT-large	8	11	+3	37.5
ROBERTa-base	5	8	+3	60
ROBERTa-large	10	12	+2	20
Electra-base	4	6	+2	50
Electra-large	9	11	+2	22.2
ModernBERT-base	3	6	+3	100
ModernBERT-large	10	12	+2	20

## Error and reasoning chain analysis

In this section we provide 2 examples using BERT-base, one where the model gets a question wrong and one where the model gets the question right.

### Case study of incorrect prediction: quantum physics problem

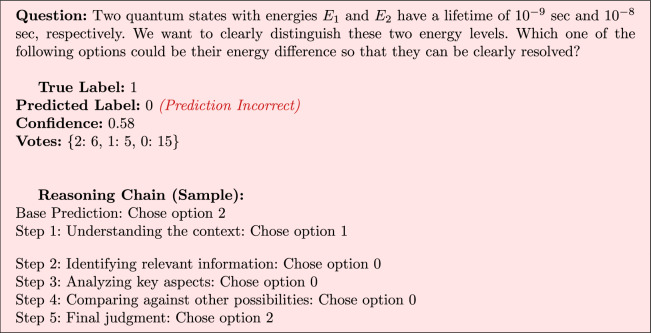



**Analysis:** This example highlights a failure case. The reasoning chain shows fluctuation between options (2 $\rightarrow$ 1 $\rightarrow$ 0 $\rightarrow$ 0 $\rightarrow$ 0 $\rightarrow$ final 2), ultimately settling on an incorrect prediction (predicted 0, final judgment 2, true 1). *Note: The final judgment in the chain (2) differs from the recorded Predicted Label (0). We analyze based on the Predicted Label 0 vs True Label 1.* The core concept is the Energy-Time Uncertainty Principle ($\Delta E \Delta t \gtrsim \hbar$). Resolvability requires the energy difference ($\Delta E$) to be significantly larger than the energy uncertainty associated with the lifetimes. The shorter lifetime ($10^{-9}$ s) imposes the stricter constraint. The reasoning steps seem erratic and don’t clearly reflect the application of this principle. The high vote count for the incorrect option 0 (15 votes) despite a moderate confidence (0.58) suggests potential issues in vote aggregation or confidence calibration.

### Case study of correct prediction: molecular symmetry problem

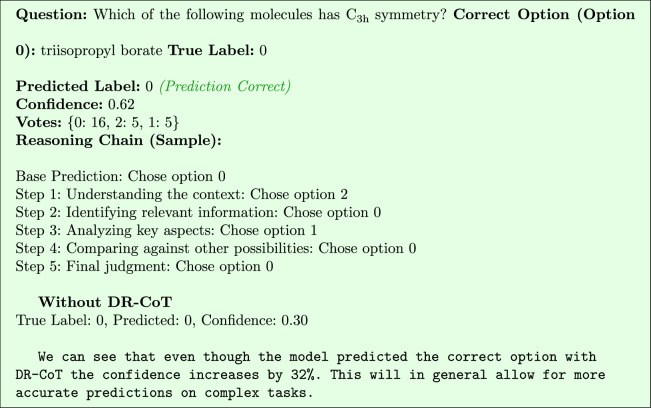



**Analysis:** A correct prediction for a molecular symmetry question. Determining point groups requires identifying symmetry elements (rotation axes, reflection planes, inversion centers). $\hbox {C}_{3h}$ symmetry requires a $C_3$ axis and a $\sigma _h$ plane (perpendicular to the $C_3$ axis). Triisopropyl borate, $\hbox {B(O-iPr)}_3$, can adopt a conformation with this symmetry (planar $\hbox {BO}_3$ core, isopropyl groups arranged propeller-like). The reasoning chain shows some fluctuation (0 $\rightarrow$ 2 $\rightarrow$ 0 $\rightarrow$ 1 $\rightarrow$ 0 $\rightarrow$ 0), but the base prediction and final judgment are correct. The high vote count for option 0 (16) aligns with the correct answer, despite the intermediate steps exploring other options. This might indicate that while exploring alternatives, the system maintained a stronger belief in the correct answer, possibly due to features matching the $\hbox {C}_{3h}$ criteria.

### Error analysis and insights for DR-CoT

*Observation:* An analysis of the reasoning chains for two distinct questions–one resulting in an incorrect physics prediction and the other in a correct chemistry prediction–reveals significant variability in the framework’s refinement process. The incorrect prediction (Base 2 $\rightarrow$ 1 $\rightarrow$ 0 $\rightarrow$ 0 $\rightarrow$ 0 $\rightarrow$ Final 2) featured an erratic reasoning chain, suggesting the refinement process failed to converge stably or effectively towards the correct answer. Conversely, the successful prediction (Base 0 $\rightarrow$ 2 $\rightarrow$ 0 $\rightarrow$ 1 $\rightarrow$ 0 $\rightarrow$ Final 0), while ultimately correct, also demonstrated notable fluctuations in its reasoning path. This indicates an inherent instability might exist within the refinement steps, even when the final outcome is accurate. Further complicating diagnosis, the repetitive nature of the step descriptions (e.g., “Understanding the context,” “Identifying relevant information”) across diverse problems limits precise insight into why refinement succeeds in some instances and fails in others.

*Insight for DR-CoT* These observations suggest that the refinement steps within DR-CoT exhibit variable effectiveness. Critically, the instability observed in the reasoning path does not strictly correlate with incorrectness, as demonstrated by the successful chemistry example. However, the failure in the physics example underscores that the mechanism guiding transitions between reasoning steps may be flawed or insufficient to reliably correct errors or navigate complex derivations. The framework could benefit significantly from more robust mechanisms ensuring refinement steps are consistently productive. *Recommended Improvements* include developing stronger self-correction capabilities, integrating explicit verification checks within each reasoning step, and potentially creating stronger constraints or heuristics to guide transitions and prevent erratic jumps in the reasoning process.

### Challenges in applying foundational concepts

*Observation:* The two analyzed questions presented distinct challenges related to applying foundational concepts. The incorrect prediction involved a quantitative physics problem requiring the application of the Energy-Time Uncertainty Principle ($\Delta E \Delta t \gtrsim \hbar$). The failure strongly implies fundamental flaws in how this principle was applied, potentially stemming from incorrect lifetime selection, miscalculation of the inherent energy uncertainty, or an improper comparison between the required energy difference for resolution and the calculated uncertainty. In contrast, the correct prediction involved a qualitative chemistry problem focused on identifying molecular symmetry elements for the $\hbox {C}_{3h}$ point group. The system successfully identified the necessary $C_3$ axis and $\sigma _h$ plane in the structure of triisopropyl borate, although the reasoning chain exhibited transient uncertainties.

*Insight for DR-CoT* The framework demonstrates mixed performance across these different reasoning domains. A notable pattern emerges suggesting greater challenges with quantitative tasks that necessitate precise numerical calculations or the strict application of mathematical inequalities, as seen in the physics problem. The framework appears relatively stronger in qualitative reasoning involving pattern matching or rule application, such as symmetry identification in chemistry.

*Recommended Enhancements* to address the weaknesses in quantitative reasoning include incorporating steps for explicit calculation verification, adding unit consistency checks, developing domain-specific reasoning procedure templates to guide complex calculations, and potentially implementing threshold-based validation mechanisms to better handle inequalities and comparisons inherent in principles like the Uncertainty Principle.

## Ablation

In Table 7 we conduct an ablation study, using different components of our proposed methodology and evaluate the impact on accuracy when evaluated on GPQA Diamond.

**Table 7:** Performance comparison of DR-CoT variants across different base models.

Model	DR-CoT (Recursion Only), (%)	DR-CoT (Voting Only), (%)	DR-CoT (Full), (%)
BERT-base	22.7	23.2	23.7
RoBERTa-base	23.7	24.2	25.8
ModernBERT-base	23.2	24.3	25.0

## Conclusion

In this work, we introduced the Dynamic Recursive Chain-of-Thought (DR-CoT) framework, which integrates recursive reasoning with dynamic context truncation and a voting mechanism to improve the performance of large language models in complex reasoning tasks. By retaining only the most relevant context within a fixed token limit and aggregating outputs from multiple independent reasoning chains. Our evaluations on challenging benchmarks such as GPQA Diamond and AIME2024 demonstrate consistent delta performance gains across diverse model architectures, underscoring the effectiveness and robustness of our approach. Moreover, experiments on the HumanEval benchmark further validate DR-CoT-s impact on code generation tasks, where DR-CoT–enabled models not only close the gap but, in many cases, outperform larger state-of-the-art models like GPT-3.5, GPT-4, and LLaMA2-70B. These results highlight the potential of DR-CoT to bridge performance disparities, enabling even parameter-efficient, BERT-sized models to deliver competitive, if not superior, performance compared to traditional large language models.

## Future work

An important direction for future work is to explore alternative strategies for the voting mechanism in our framework. While our current approach employs a fixed number of reasoning chains, this can lead to significant computational overhead, particularly when the complexity of the task does not warrant such extensive processing.

One promising avenue is the development of adaptive voting schemes that dynamically adjust the number of reasoning chains based on task complexity. For example, the system could estimate task difficulty through initial confidence scores or uncertainty measures, and subsequently allocate more reasoning chains to challenging questions while reducing the number for simpler instances. This adaptive strategy could maintain, or even enhance, overall accuracy while reducing computational cost. Additionally, we plan to investigate lightweight approximations and model pruning techniques to further streamline the voting process. These methods have the potential to make our DR-CoT framework more scalable and efficient for real-world applications, where computational resources are often limited. By incorporating these adaptive mechanisms, we aim to achieve a more balanced trade-off between computational efficiency and reasoning accuracy.

## Limitations

Despite the promising results, our approach has several limitations:

- *Information Loss* The dynamic context truncation mechanism, while efficient, may inadvertently discard relevant information, particularly in tasks where subtle reasoning steps are critical.
- *Computational Overhead* The use of multiple independent reasoning chains for the voting mechanism increases computational costs (both in terms of time and memory), which might be prohibitive in real-time or resource-constrained environments.
- *Vulnerability to Systemic Bias* The voting mechanism is effective at mitigating random, independent errors but remains vulnerable to systemic biases in the foundation model. If the model has an inherent bias, all reasoning chains may converge on the same incorrect answer, a scenario that voting cannot correct.

Addressing these limitations remains an important direction for future work, as we strive to further optimize the balance between efficiency, robustness, and reasoning depth in advanced language models.

## Footnotes

1 https://huggingface.co/datasets/HuggingFaceH4/aime_2024

2 The models in the table are not necessarily state-of-the-art; we selected those with which our DR-CoT-enabled models are competitive or superior.
